# Deaths from Anæsthetics

**Published:** 1898-01-29

**Authors:** 


### DEATHS FROM ANÆSTHETICS.

During the past year we have endeavoured to obtain information in regard to all the deaths which have occurred in consequence of or during the administration of anæsthetics. The results of our inquiry are startling and distressing.

On March 27th we said that already twenty-two deaths from chloroform had occurred; on April 24th we recorded five more; on June 12th twelve more; on August 14th we gave a list of ten cases of deaths from anæsthetics, and now we take up the sad story once again. It is too long to give in its entirety this week, but we hope next week to finish the series. We give in each case the date of the inquest, the authority for the report, and any particulars of interest which we have been able to ascertain. We would, however, wish this to be understood, that we have in this matter—a matter of very serious public concern—appealed entirely to public records, and that there are circumstances which make us feel somewhat doubtful whether all the cases that have occurred have ever obtained publicity. While, then, we give chapter and verse for those we publish, we would say that in number they constitute the very minimum, and that it is quite possible that many other deaths from anæsthetics may have occurred which have never been published.

The *Brighton Gazette*, July 24th, reports an inquest on a woman who died at the Lying-in Institution while ether was being administered for an operation for internal hæmorrhage.

The *Liverpool Post*, July 27, reports an inquest on a man, aged 36, who died at the Birkenhead Workhouse under the influence of chloroform. Just as the operation began the breathing ceased.

The *Southern Times*, July 31st, reports an inquest on a baby who died at the Yeatman Hospital under chloroform directly he came under its influence.

The *Nottingham Daily Express*, August 2nd, reports an inquest on a man, aged 20, who died at the General Hospital while under the influence of chloroform. The case was one of pleuritic effusion and dropsy.

The *Western Morning News*, August 2nd, reports the death from chloroform of a man, aged 53, at the Boscombe Cottage Hospital.

The *Evening Standard*, August 7th, reports an inquest on a lady, aged 61, in London (Miss Elliott), while being operated on by a gynæcologist, the anæsthetic being gas, followed by ether. The operation was a prolonged one, and the death was attributed primarily to the operation itself, and only in a secondary degree to the anæsthetic.

The *Midland Evening News*, August 14th, reports an inquest held on a woman, aged 33, who died at the Wolverhampton Women's Hospital whilst being placed under chloroform. She died suddenly, before the operation was commenced, about a minute after the administration was commenced.

The *Daily News*, August 16th, reports an inquest on a man, aged 32, who died at the Royal Naval Hospital, Chatham, while under the influence of chloroform.

The *Liverpool Daily Courier*, August 16th, reports an inquest at Rhyl on a woman, aged 29, who died under chloroform, administered for tooth extraction, in a chair.

The *Sun*, August 25th, reports an inquest on a youth, aged 17, who died under chloroform at the London Temperance Hospital. The chloroform was administered for tooth extraction, and about half a dozen stumps had been removed when death took place.

The *Nottingham Daily Express*, August 26th, reports an inquest on a child, aged three years, who died at the Nottingham Children's Hospital whilst under chloroform, for the opening of an abscess in the thigh. It was stated that the respiration suddenly stopped, but that the pulse did not cease beating until half an hour afterwards.

The *East Anglian Times*, September 4th, reports an inquest on a son of the President of Queen's College, Cambridge, which took place after chloroform had been administered, for the removal of adenoids.

The *Standard*, September 7th, reports an inquest on a man, aged 39, who died in the Liverpool Royal Infirmary. Chloroform was being administered, but the patient died before the operation was commenced.

(*To be continued.*)

